# Quantifying and modeling fabric surface roughness discrepancy: Consistency between physical and digital textures in online shopping

**DOI:** 10.1371/journal.pone.0352028

**Published:** 2026-06-18

**Authors:** Eugene Lee, Youngjoo Chae

**Affiliations:** 1 Department of Clothing & Textiles, Yonsei University, Seoul, Republic of Korea; 2 Department of Clothing & Textiles, Chungbuk National University, Cheongju, Republic of Korea; King Mongkut’s University of Technology North Bangkok, THAILAND

## Abstract

In digital textile commerce, the absence of tactile interaction limits consumers’ ability to perceive fabric properties, often leading to mismatched expectations and product dissatisfaction. This study aimed to quantify the perceptual discrepancy in surface roughness (RDP) between physical fabrics and their digital representations and to identify structural parameters that influence this discrepancy. Plain- and twill-woven fabric specimens were prepared with varying densities, weights, and thicknesses. Surface roughness of physical fabrics was measured using atomic force microscopy (AFM), while digital roughness values were extracted from scanned images using ImageJ. Statistical analyses, including correlation and regression modeling, were applied to identify key predictors of RDP. Results showed that in plain-woven fabrics, lower weft density and higher warp density under fixed fabric weight conditions yielded the lowest RDP values, whereas in twill-woven fabrics, the perceptual gap was minimized at higher fabric weights and lower weft densities. These findings provide practical insights for improving visual–tactile alignment in virtual textile presentation and can inform fabric structural design for enhanced accuracy in online representation. Future research may explore nonlinear modeling and multisensory feedback systems to further reduce perceptual discrepancies.

## 1. Introduction

When consumers evaluate textile and apparel products, comfort is consistently recognized as a key determinant of purchase decisions [1]. Comfort is not a single attribute but a multifaceted concept arising from the interaction between the human body and textile materials in the context of clothing. Previous studies have described comfort in clothing can be categorized into three primary dimensions: thermophysiological comfort, tactile comfort, and movement comfort. Among these, tactile comfort pertains to sensory perceptions associated with the fabric’s contact with the skin of human, encompassing attributes such as irritation, prickle, and softness or roughness. Since tactile comfort directly interacts with the skin, fabrics with coarse textures may induce discomfort and, in severe cases, trigger allergic reactions or dermatological conditions. In contrast, fabrics with a soft texture contribute to a sense of comfort and psychological well-being, enhancing the wearer’s overall mood. Thus, tactile comfort refers to the property of a textile that ensures a non-irritating, soft, and pleasant tactile sensation upon skin contact, and it is primarily influenced by factors such as smoothness, roughness, and friction of the fabrics.

Surface roughness describes the degree of microscopic unevenness present on a material surface and reflects variations in surface geometry at a fine scale [2]. In textile materials, these surface characteristics arise from complex interactions among fiber and yarn properties as well as fabric structural parameters [1]. Factors such as fiber fineness and morphology, yarn twist and arrangement, and the interlacing of warp and weft yarns collectively contribute to the formation of surface asperities. In addition, fabric-level parameters—including weave type, fabric density, weight, and thickness—play an important role in shaping the surface profile of woven textiles. Consequently, fabric surface roughness is not determined by a single parameter but emerges from the combined effects of material composition and structural configuration [1,3,4].

With the rapid growth of digital commerce environments, apparel purchasing has increasingly shifted toward online platforms. Advances in digital technologies and the diversification of e-commerce services have expanded consumers’ access to a wide range of textile products without spatial or temporal constraints [5–7]. Despite these advantages, the majority of consumers still prefer the offline shopping environment over the online one. This preference is largely due to the tactile experience provided by offline shopping, where consumers can directly observe and touch products to assess their color, material, and texture. For items such as clothing and shoes, customers can also try them on to determine the right size. The lack of physical interaction with products prior to purchase in the online shopping environment is cited as one of the primary factors contributing to the continued preference for offline stores [8–12].

Sensory perception plays a central role in how consumers evaluate apparel products during the purchasing process [8,13]. Among various sensory modalities, visual information typically serves as the initial channel through which consumers form expectations about a product, influencing judgments related to appearance and perceived quality. However, visual cues alone are often insufficient for fully assessing textile materials. Tactile perception provides complementary information that enables consumers to evaluate material characteristics such as surface texture, compliance, and overall fabric feel. Together, visual and tactile inputs contribute to a more comprehensive understanding of apparel products, shaping both perceived quality and purchase confidence.

In online shopping environments, consumers evaluate textile products primarily through visual information, without direct physical interaction. As a result, surface-related tactile attributes such as roughness, softness, weight, or elasticity cannot be directly perceived. Among these attributes, the hand of the fabric—the subjective sensation experienced through touch [14,15]—plays a critical role in evaluating clothing and bedding materials. The absence of direct tactile interaction therefore presents a fundamental challenge for accurately conveying material properties in online retail contexts. To compensate for this limitation, online retailers commonly provide descriptive texture information (e.g., “soft suede-like feel” or “silky smooth texture”) or high-resolution close-up images intended to support indirect assessment of surface characteristics. However, such visual or textual cues remain insufficient to fully convey tactile sensations, as current digital representations cannot replicate physical attributes such as touch, flexibility, thickness, or weight perception. This limitation is particularly pronounced for clothing and textile products, which are typically presented in two-dimensional formats online, making it difficult for consumers to intuitively judge surface roughness. Previous research has reported substantial discrepancies between offline and online evaluations of fabric roughness. Specifically, the accuracy of consumer roughness grading was reported to be 71% ± 15.1 in offline settings, compared to 48% ± 12.9 in online environments [14]. Such discrepancies may contribute to consumer dissatisfaction and product returns after purchase, with potential long-term impacts on brand perception [16]. These findings highlight the difficulty of accurately assessing textile roughness based solely on visual information and underscore the need for more specific and objective information regarding fabric surface roughness in online shopping environments.

Previous studies have attempted to compensate for the absence of direct tactile experience in online shopping environments by enhancing visual or cognitive cues. These studies commonly suggest that consumers rely on visual information, prior tactile experience, and mental imagery to infer material properties when physical contact is unavailable [17,18]. In this context, tactile mental imagery has been proposed as a mechanism through which consumers integrate visual stimuli with past sensory experience to evaluate product quality [17], while other studies have examined how prior tactile experience supports visual assessment of material properties without direct touch [18]. In addition, research based on consumer behavior frameworks has investigated how different online presentation formats influence perceived tactile sensations. For example, the Stimulus–Organism–Response (SOR) paradigm has been applied to analyze how static images, zoomed-in views, and videos affect consumers’ tactile impressions in online retail settings [19]. Related work has also examined the consistency of perceived fabric roughness between online and offline environments [14]. While these studies provide valuable insights into perceptual mechanisms and consumer responses, they primarily rely on subjective evaluations obtained from human participants. As a result, it remains difficult to derive objective, quantifiable measures that directly capture discrepancies between digitally represented textures and physically experienced surface roughness.

Taken together, prior studies have primarily sought to mitigate the absence of tactile experience in online shopping by enhancing visual or perceptual cues. While such approaches contribute to understanding how consumers infer material properties from images or videos, they remain limited in their ability to convey actual tactile sensations. Visual representations may support approximate judgments of texture, but they do not provide direct information about physical surface characteristics. Moreover, much of the existing literature is based on subjective evaluations obtained from human participants. Although some consumers may successfully infer material properties from visual cues, others remain uncertain without physical interaction, resulting in variability across individuals. Consequently, these approaches offer limited accuracy and predictive consistency. In contrast, the present study adopts a quantitative perspective by directly measuring surface roughness and examining discrepancies between physically measured and digitally represented textures. By introducing an objective metric to capture the difference between actual fabric roughness and image-based roughness, this study aims to assess the extent of visual–tactile inconsistency in digital textile representation. Furthermore, the study investigates how structural fabric parameters—including weave type, fabric weight, and fabric density—contribute to this discrepancy.

## 2. Results

### 2.1. Analysis of warp and weft densities based on FE-SEM images

**Fig 1:**
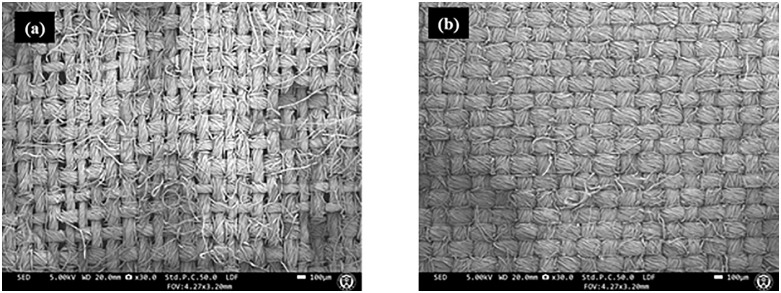
Representative FE-SEM images used to quantify warp and weft yarn densities (EPI and PPI) in woven fabric specimens. Images illustrate typical surface morphology of plain-woven samples: **(a)** P1 and **(b)** P3.

**Fig 2:**
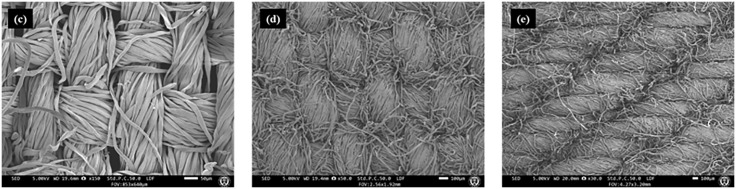
FE-SEM surface images of selected specimens illustrating variations in yarn number and fabric density: (c) P1, (d) P5, and (e) T4.

**Fig 3:**
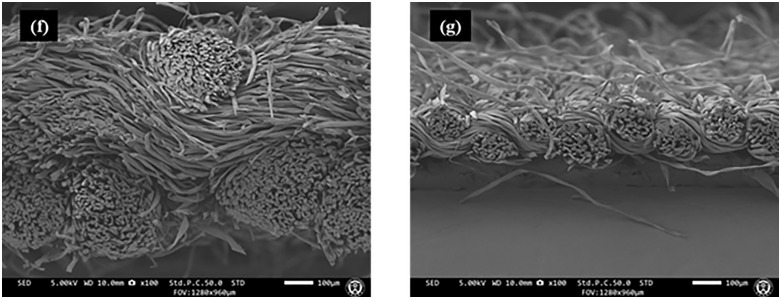
Cross-sectional FE-SEM images used for fabric thickness measurement: (f) T4 and (g) P2. Thickness values were obtained from repeated measurements across the cross-section.

In this study, FE-SEM was employed to capture SEM images of various plain and twill woven fabrics for the purpose of analyzing fabric density. Fig 1 presents representative SEM images used to measure the density of the specimens. The calculated warp and weft densities (EPI and PPI) are summarized in Table 7 in the beginning of this Methods section. Among the specimens, several characteristic cases are noteworthy (Fig 2). Specimen P1 exhibited a high density of 159 EPI and 110 PPI, yet it was the lightest in weight and had the thinnest yarns, resulting in visible spacing between yarns (Fig 2(c)). In contrast, P5 had the lowest warp density among the nine specimens but showed the second heaviest fabric weight following specimen T4. This is likely due to its use of the thickest yarns, which aligns with the observation that thicker yarns tend to increase fabric weight while reducing thread density (Fig 2(d)). Fig 2(e) shows the SEM image of specimen T4, which had a relatively high weft density, the thickest yarns, and the greatest overall fabric weight among all specimens. Additionally, the fabric thickness was measured through cross-sectional image analysis using FE-SEM. As shown in Fig 3, cross-sections of the fabrics were imaged, and the thickness was measured repeatedly. For each specimen, five repeated measurements were performed to obtain an average thickness value, which is reported in Table 7. Among the nine specimens, the thickest fabric was T4 (784.53 μm), while the thinnest was P2 (188.80 μm).

### 2.2. Comparison of surface roughness profiles between actual fabrics and scanned fabric images

**Fig 4:**
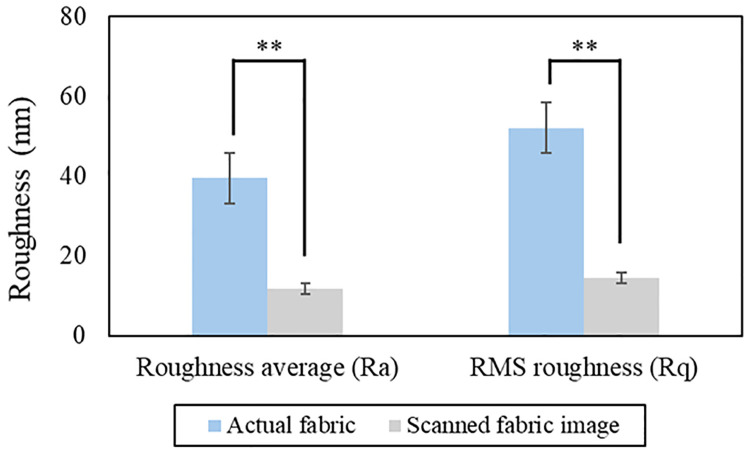
Comparison of surface roughness between actual fabrics and scanned fabric images, expressed as roughness average (Ra) and root mean square roughness (Rq). Error bars indicate standard deviations. A statistically significant difference was observed between groups (**p < .01).

As shown in Table 1, the surface roughness of the actual fabric samples ranged from 16.62 to 79.93 nm for Ra and from 21.81 to 107.25 nm for Rq. In contrast, the roughness values of the scanned samples ranged from 4.01 to 17.35 nm for Ra and from 9.74 to 85.17 nm for Rq, generally exhibiting substantially lower values than the actual fabrics. This result suggests that visual information alone may not sufficiently capture the fine surface irregularities of textile materials. To statistically assess these differences, an independent samples t-test was performed. The results (Fig 4) showed a statistically significant difference between the two groups, with a p-value of 0.003 at the 0.05 significance level. This indicates a clear perceptual discrepancy in surface roughness between actual fabrics and their scanned image representations. Accordingly, the present study proceeds to investigate how structural characteristics—such as weave type, thickness, weight, and density—may contribute to this observed discrepancy.

**Table 1:** Surface roughness parameters of actual fabrics and scanned fabric images, and the corresponding roughness discrepancy percentage (RDP). Roughness average (Ra) and root mean square roughness (Rq) were used to quantify physical–digital discrepancies.

Specimen name	Surface roughness of the actual fabric		Surface roughness of the scanned fabric image		Roughness discrepancy percentage (RDP)	
	Roughness average, Ra (nm)	Root Mean Square (RMS) Roughness, Rq (nm)	Roughness average, Ra (nm)	Root Mean Square (RMS) Roughness, Rq (nm)	RDP of Ra (%)	RDP of Rq (%)
**P1**	56.05	74.35	4.01	69.39	92.85	6.67
**P2**	42.38	55.42	4.85	49.40	88.56	10.86
**P3**	43.90	58.56	9.98	46.17	77.27	21.16
**P4**	16.62	21.81	9.86	9.74	40.67	55.34
**P5**	79.93	107.25	17.35	85.17	78.29	20.59
**T1**	21.01	29.00	12.15	14.74	42.17	49.17
**T2**	26.85	35.60	16.95	15.43	36.87	56.66
**T3**	33.86	43.46	16.96	22.61	49.91	47.98
**T4**	35.00	43.59	13.81	26.38	60.54	39.48

### 2.3. Effect of fabric weave type on surface roughness discrepancy

An independent samples t-test was conducted to examine the difference in surface roughness discrepancy between plain-woven and twill-woven fabrics. As shown in Table 2, the result was statistically significant with a t-value of 3.494 and a p-value of 0.005, indicating that the discrepancy between the two fabric structures was significant at the 0.05 level. Additionally, correlation analyses were performed to investigate the relationship between surface roughness discrepancy and fabric thickness, weight, and density, regardless of weave type. The results revealed generally low correlation coefficients and p-values exceeding 0.05, suggesting that these factors do not have a statistically significant effect on roughness discrepancy. These findings imply that the weave structure itself may serve as a primary factor influencing surface roughness discrepancy. Accordingly, the present study proceeds with separate analyses for plain- and twill-woven fabrics to better reflect the structural differences between these fabric types.

**Table 2:** Comparison of surface roughness discrepancy between plain-woven and twill-woven fabrics based on independent samples t-test. Values are presented as mean ± standard deviation.

Variables	Mean	Std. dev.	*t*-value
Surface roughness discrepancy of plain-woven fabrics	45.27	24.66	–
Surface roughness discrepancy of twill-woven fabrics	17.00	6.10	3.494***

***p < .001

### 2.4. Influence of thickness, weight, and density on discrepancy in plain-woven fabrics

To investigate the relationship between fabric structure and roughness discrepancy (RDP) in plain-woven fabrics, Pearson correlation analysis was conducted using fabric thickness, weight and fabric density as independent variables. As presented in Table 3, both Ra- and Rq-based RDP values showed statistically significant correlations with fabric thickness, weight, and warp yarn density, while weft yarn density exhibited no significant correlation with either parameter. Specifically, cross-sectional thickness and fabric weight were positively correlated with RDP, indicating that fabrics with greater thickness and weight tend to exhibit larger discrepancies between the actual and image-based surface roughness. In contrast, warp density showed a significant negative correlation with RDP (Ra: r = –.490; Rq: r = –.509; p < .01), suggesting that lower warp densities—by increasing the spacing between yarns and thus introducing more prominent surface irregularities—may lead to higher perceptual discrepancies. Interestingly, weft yarn density was not significantly correlated with roughness discrepancy. This may be attributed to the structural characteristics of plain-woven fabrics, in which warp yarns dominate the surface texture due to their higher interlacing frequency and alignment direction.

**Table 3:** Pearson correlation coefficients between fabric characteristics (thickness, weight, weft density, and warp density) and surface roughness discrepancy in plain-woven fabrics.

		Fabric characteristics			
Surface Roughness Discrepancy Metric		Thickness by cross-sectional	Weight	Weft yarn density	Warp yarn density
**Ra**	PearsonCorrelation	.424**	.130**	−.020	−.490**
	*p*-value	**.000**	**.000**	.535	**.000**
**Rq**	Pearson Correlation	.450**	.157**	−.045	−.509**
	*p*-value	**.000**	**.000**	.159	**.000**

**Note**. *p*-values were calculated using two-tailed Pearson correlation analysis.

**p < .01

To examine the interaction effects among fabric characteristics, a multivariate analysis of variance (MANOVA) was performed, followed by Duncan’s post hoc test. The results revealed no statistically significant interaction effects among the independent variables—namely, fabric thickness, weight, warp density, and weft density (*p* > .05). This suggests that these fabric properties act independently, rather than influencing one another, in their contribution to surface roughness discrepancy. These findings are in agreement with previous study [20], which also reported that structural fabric parameters tend to affect surface characteristics individually rather than through mutual interaction. Accordingly, subsequent analyses in this study focused on modeling the individual effects of each variable through a multiple regression approach.

### 2.5. Influence of thickness, weight, and density on discrepancy in twill-woven fabrics

Subsequently, the relationship between fabric properties and surface roughness discrepancy (RDP) was analyzed for twill-woven fabrics. As shown in Table 4, all structural parameters—including fabric thickness, weight, warp density, and weft density—exhibited statistically significant positive correlations with RDP (*p* < .01). This suggests that as a twill fabric becomes thicker, heavier, and denser, the discrepancy between the actual and image-based perception of surface roughness tends to increase. Notably, unlike plain-woven fabrics, twill-woven fabrics showed a significant correlation between weft density and RDP. This difference can be attributed to the structural characteristics of twill weaves, which include longer floats and more complex interlacing patterns. As the weft density increases, the yarn spacing decreases and the fabric structure becomes finer, potentially amplifying the perceived discrepancy between visual and physical roughness. Matusiak & Kosiuk (2025) [21] also reported that twill fabrics possess more complex and irregular constructions compared to plain weaves, and that these structural differences significantly influence tactile comfort and surface characteristics. In contrast, plain-woven fabrics, due to their uniform structure and higher interlacing frequency, tend to be less affected by changes in weft density. Consequently, warp density plays a more dominant role in determining surface roughness discrepancy in plain weaves, whereas both warp and weft densities are influential in twill fabrics.

**Table 4:** Pearson correlation coefficients between fabric characteristics (thickness, weight, weft density, and warp density) and surface roughness discrepancy in twill-woven fabrics.

		Fabric characteristics			
Surface Roughness Discrepancy Metric		Thickness by cross-sectional	Weight	Weft yarn density	Warp yarn density
**Ra**	PearsonCorrelation	.913**	.921**	.761**	.647**
	*p*-value	**.000**	**.000**	**.000**	**.000**
**Rq**	Pearson Correlation	.898**	.908**	.771**	.629**
	*p*-value	**.000**	**.000**	**.000**	**.000**

**Note**. *p*-values were calculated using two-tailed Pearson correlation analysis.

**p < .01

To further investigate the structural interactions among variables in twill-woven fabrics, a multivariate analysis of variance (MANOVA) was conducted, followed by Duncan’s post hoc test. The results showed no statistically significant interaction effects among thickness, weight, warp density, and weft density (*p* > .05). This indicates that, as in the case of plain-woven fabrics, the structural parameters in twill fabrics operate independently, with each variable contributing individually to surface roughness discrepancy (RDP) rather than through interdependent effects. These findings are consistent with those observed in plain-woven fabrics, suggesting a general pattern across weave types in which fabric characteristics influence RDP primarily through their individual effects. Accordingly, the development of regression models in the subsequent analysis is focused on assessing the independent contribution of each structural factor.

### 2.6. Development of a predictive model for reducing surface roughness discrepancy

To develop a predictive model for reducing surface roughness discrepancy (RDP), a stepwise multiple regression analysis was conducted. As shown in Table 5, for plain-woven fabrics, fabric weight, warp yarn density, and weft yarn density were identified as significant predictors of RDP. All predictors exhibited negative regression coefficients, indicating that higher fabric weight and density contribute to a decrease in RDP. This result suggests that heavier and denser fabrics tend to have more uniform and refined surface characteristics, which in turn minimize the discrepancy between the roughness of the scanned image and that of the actual fabric. Ghaderpanah & Ghareaghaji (2015) [20] demonstrated that these structural parameters can be effectively utilized in predictive models of perceived fabric roughness.

**Table 5:** Stepwise multiple regression analysis for predicting surface roughness discrepancy in plain-woven fabrics using fabric structural parameters. Models are reported separately for Ra- and Rq-based RDP.

Model	Predictors	Unstandardized Coefficients		Adjusted R-square	*t-*value	p-value
		Beta (β)	SE			
Dependent Variable: Roughness average (Ra) of plain-woven fabric						
1	(Constant)	384.661	2.991	.994	128.591	<.001
	Weft yarn density	−3.350	.026		−127.404	
	Weight	−324.739	2.952		−110.010	
	Warp yarn density	−1.585	.019		−83.702	
Dependent Variable: RMS Roughness (Rq) of plain-woven fabric						
1	(Constant)	516.610	3.759	.950	137.425	<.001
	Weft yarn density	−4.498	.033		−136.116	
	Weight	−433.444	3.710		−116.843	
	Warp yarn density	−2.150	.024		−90.319	

In the case of twill-woven fabrics (Table 6), weft yarn density and fabric weight emerged as significant predictors. Here, weft density showed a positive relationship with RDP, while weight maintained a negative association. This indicates that twill fabrics with lower weft density and higher weight are more likely to present a visually consistent surface structure, thus reducing the gap between visually perceived and physically measured roughness. Such behavior may be attributed to the complex interlacing and longer floats characteristic of twill weaves, which render the surface more sensitive to changes in weft yarn density [21].

**Table 6:** Stepwise multiple regression analysis for predicting surface roughness discrepancy in twill-woven fabrics using fabric structural parameters. Models are reported separately for Ra- and Rq-based RDP.

Model	Predictors	Unstandardized Coefficients		Adjusted R-square	*t-*value	p-value
		Beta (β)	SE			
Dependent Variable: Roughness average (Ra) of twill-woven fabric						
1	(Constant)	7.494	.176	.940	42.585	<.001
	Weft yarn density	37.275	.401		93.027	
	Weight	−.352	.009		−38.968	
Dependent Variable: RMS Roughness (Rq) of twill-woven fabric						
1	(Constant)	13.681	.193	.923	70.994	<.001
	Weft yarn density	36.239	.439		82.592	
	Weight	−.354	.010		−35.814	

**Fig 5:**
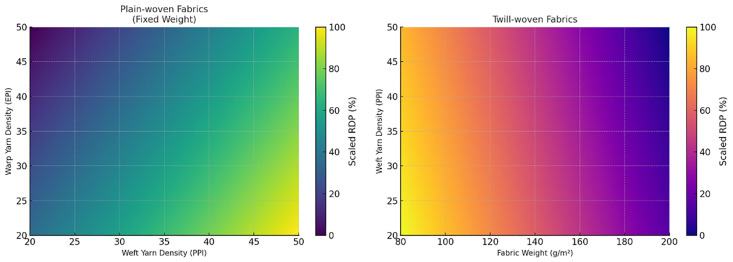
Predicted surface roughness discrepancy (RDP) conditions derived from regression models. Heatmaps illustrate regions of minimized RDP: (left) plain-woven fabrics at a fixed weight of 202 g/m² as a function of warp and weft yarn densities (EPI, PPI); (right) twill-woven fabrics as a function of fabric weight (g/m²) and weft yarn density (PPI).

Based on the developed predictive models, the structural conditions associated with minimized surface roughness discrepancy (RDP) were identified and visualized using heatmaps (Fig 5). For plain-woven fabrics, under a fixed fabric weight of 202 g/m², the RDP was predicted to be at its lowest when the warp yarn density exceeded 45 EPI and the weft yarn density remained below 25 PPI. For twill-woven fabrics, the lowest RDP values were observed when the fabric weight was above 180 g/m² and the weft yarn density was below 25 PPI.

## 3. Discussion

This study quantified the perceptual discrepancy in surface roughness (RDP) between physical fabrics and their digital image representations, and investigated the influence of structural parameters through predictive modeling. Multiple regression analysis and heatmap visualizations confirmed that fabric weight, warp yarn density, and weft yarn density significantly affect RDP, with the impact varying by weave type. For plain-woven fabrics, under a fixed fabric weight of 202 g/m², the RDP was minimized when the warp yarn density exceeded 45 EPI and the weft yarn density was below 25 PPI. The lower RDP observed for specific density combinations in plain-woven fabrics is interpreted as resulting from the interaction between structural parameters and surface-related visual and tactile cues. Higher warp yarn density increases yarn intersection frequency, leading to a more homogeneous surface structure. This structural uniformity produces relatively consistent patterns in both tactile roughness measured by AFM and image-based roughness derived from scanned images. Conversely, when weft yarn density is not excessively high, fine surface asperities between yarns are reduced, which helps mitigate visual–tactile discrepancies. These effects are further mediated by surface micro-geometry and reflectance characteristics, which influence visual texture perception. In twill-woven fabrics, the lowest RDP values were predicted when the fabric weight was above 180 g/m² and the weft yarn density was less than 25 PPI. These findings offer practical design guidelines for optimizing digital textile representation in online platforms. By carefully adjusting structural parameters, the alignment between visual and tactile perceptions can be improved, potentially reducing product returns and enhancing consumer satisfaction in virtual shopping environments. Several limitations should be noted. The sample size was relatively limited, which may constrain statistical power and the generalizability of the regression results. Accordingly, the findings should be interpreted as exploratory, focusing on identifying relative trends in RDP associated with variations in structural parameters rather than establishing definitive predictive models. In addition, stepwise regression is sensitive to sample size and model assumptions, and thus the regression outcomes should be interpreted with caution. Building on these findings, future research could extend the present framework by incorporating a broader range of fabric types (e.g., knitted or functional textiles) and adopting nonlinear modeling approaches. In particular, machine learning–based regression methods—such as Gaussian process regression, support vector regression, or ensemble-based models—may be well suited to capturing complex, non-additive relationships between fabric structural parameters and perceptual discrepancies. Recent work by Lee et al. (2025) [22] demonstrated that tactile roughness perception can be predicted using machine learning models trained on multidimensional mechanical properties, while also emphasizing the importance of rigorous cross-validation and the challenges associated with limited sample sizes and model generalizability. These observations are consistent with the exploratory scope of the present study and motivate future validation using larger and more diverse datasets. Furthermore, predicted tactile attributes derived from such models could potentially be integrated into multisensory simulation systems, such as haptic display interfaces, in which tactile feedback is rendered alongside visual fabric representations. Such multisensory approaches may contribute to reducing visual–tactile perceptual discrepancies and enhancing the sensory fidelity of digital fabric simulation environments.

## 4. Methods

### 4.1. Samples

In this study, plain- and twill-woven fabrics were selected as experimental samples to quantitatively analyze the variation in surface roughness according to fabric structure. Previous research indicates that twill weaves tend to exhibit greater surface irregularity and roughness due to their structural characteristics—namely, fewer interlacing points and longer floats—compared to plain weaves [23]. Such differences make these two weaves ideal for examining the discrepancy between visually perceived and tactilely experienced fabric textures. Additionally, Ezazshahabi et al. (2017) [24] demonstrated that the distribution of floats and surface characteristics across different weave structures significantly influence roughness values. Accordingly, plain and twill weaves are deemed suitable for comparing tactile information delivery and perceptual gaps in digital shopping environments. A total of eight specimens, consisting of plain and twill weaves with varying structural parameters, were utilized in this study. Their fundamental specifications are summarized in Table 7.

**Table 7:** Structural specifications of fabric specimens, including fiber content, weave type, yarn number, thickness, weight, and fabric density (EPI, PPI).

Specimen name	Fiber content	Weave type	Yarn number, ″	Thickness by cross-sectional, μm	Weight, g/m^2^ (gsm)	Fabric density	
						Warp yarn density, EPI	Weft yarn density, PPI
**P1**	Cotton 100%	Plain	80	198.93	68	159	110
**P2**		Plain	60	188.80	104	121	169
**P3**		Plain	40	275.73	132	114	135
**P4**		Plain	30	284.80	180	140	76
**P5**		Plain	10	563.20	272	44	68
**T1**		Twill	40	417.60	128	83	229
**T2**		Twill	30	491.20	160	76	161
**T3**		Twill	20	540.87	196	127	445
**T4**		Twill	10	784.53	336	70	206

### 4.2. Fabric characterization and roughness measurement

The weight of the specimens was measured using an electronic balance (Ohaus Explorer, OHAUS EX224G, 0.1 μg resolution, USA). The thickness and fabric density—expressed in ends per inch (EPI) and picks per inch (PPI)—were determined through image analysis using a field emission scanning electron microscope (FE-SEM, JEOL JSM-IT800, Japan).

The evaluation of fabric surface roughness can be categorized into contact and non-contact methods, with a variety of instruments and technologies developed and utilized for this purpose [1,25–27]. In this study, the surface roughness of actual fabrics was measured using a contact method with an atomic force microscope (AFM, NX-10, Park Systems, South Korea), where a probe scanned the fabric surface to detect topographical irregularities. To minimize variations in surface roughness values arising from measurement scale, all AFM measurements were fully standardized across samples. All fabric specimens were measured in contact mode using the same cantilever (PRC13) and identical scanning parameters. A fixed scan area of 3 µm × 3 µm with a resolution of 256 × 256 pixels was applied for all measurements, with a constant scan rate of 1 Hz. Each specimen was measured five times under identical conditions, and the average values were used for subsequent roughness analysis. This standardized measurement protocol was adopted to ensure consistency, reproducibility, and comparability of surface roughness data across all samples. To assess the visual perception of roughness in digital environments, scanned images of the same fabrics were analyzed using ImageJ software to extract image-based roughness values. To ensure consistency in digital image–based roughness analysis, all fabric specimens were scanned using the same equipment under identical imaging conditions. Lighting conditions, image resolution, and magnification were strictly fixed for all scans. Image processing and roughness extraction were performed using ImageJ with identical procedures and parameter settings. Each fabric specimen was scanned and analyzed five times under the same controlled conditions, and the resulting roughness values were averaged to minimize variability arising from image acquisition.

The discrepancy between the measured and visual roughness was then quantified using the Roughness Discrepancy Percentage (RDP), calculated as follows:


$$\begin{array}{c} RDP(\%)=\frac{\left|R_{actual}-R_{scanned}\right|}{R_{actual}}\times \text{1}\text{0}\text{0} \end{array}$$ (1)

Where R_actual_ refers to the measured roughness of the physical fabric specimen, and R_scanned_ denotes the roughness value extracted from the scanned fabric image.

A higher RDP value indicates a greater discrepancy between the physical and visual roughness perceptions. An RDP of 0% implies that the roughness values of the actual and scanned specimens are identical, reflecting no perceptual discrepancy.

### 4.3. Statistical analysis

To analyze the calculated roughness data, various statistical techniques were employed. First, an independent samples t-test was conducted to examine whether there was a statistically significant difference between the measured surface roughness of the actual fabric and that obtained from the scanned images. The t-test was also used to assess whether the degree of roughness discrepancy varied depending on the type of fabric weave. To identify factors influencing roughness discrepancy, Pearson’s correlation analysis was performed between fabric characteristics—including thickness, weight, and thread density—and the measured roughness values. Additionally, multi-way analysis of variance (MANOVA) and Duncan’s post-hoc test were used to investigate potential interaction effects among the independent variables. Finally, stepwise multiple regression analysis was conducted to develop a predictive model for minimizing roughness discrepancy. All statistical analyses were carried out using MATLAB (R2021b) and SPSS (IBM SPSS Statistics ver.28) software.

## References

[1] Beyene KA, Kumelachew DM. An investigation of the effects of weave types on surface roughness of woven fabric. Textile Research Journal. 2022;92(13–14):2276–84. doi: 10.1177/00405175211010683

[2] Mooneghi SA, Saharkhiz S, Varkiani SMH. Surface roughness evaluation of textile fabrics: a literature review. Journal of Engineered Fibers and Fabrics. 2014;9(2). doi: 10.1177/155892501400900201

[3] Akgun M. The effect of fabric balance and fabric cover on surface roughness of polyester fabrics. Fibers Polym. 2013;14(8):1372–7. doi: 10.1007/s12221-013-1372-0

[4] Akgun M. Assessment of the surface roughness of cotton fabrics through different yarn and fabric structural properties. Fibers Polym. 2014;15(2):405–13. doi: 10.1007/s12221-014-0405-7

[5] Jiang Y, Lai PL, Yang CC, Wang X. Exploring the factors that drive consumers to use contactless delivery services in the context of the continued COVID-19 pandemic. J Retail Consum Serv. 2023;72:103276. doi: 10.1016/j.jretconser.2023.103276

[6] Mardosaite V, Jasinskas E, Romeika G. The transformation of digital innovative services in retail trade due to the COVID-19 pandemic: a systematic review. AE. 2024;26(67):885. doi: 10.24818/ea/2024/67/885

[7] Singh NT, Singh S, Singh S, Arora A, Dhaundiyal A, Narang A. Transforming E-Commerce: Augmented Reality (AR) and Virtual Reality (VR) Integration for Interactive and Immersive Shopping Experiences. In: 2023 7th International Conference on Electronics, Materials Engineering & Nano-Technology (IEMENTech), 2023. 1–5. doi: 10.1109/iementech60402.2023.10423551

[8] Leng X, Zhou X, Wang S, Xiang Y. Can visual language convey tactile experience? A study of the tactile compensation effect of visual language for online products. Front Psychol. 2022;13:1034872. doi: 10.3389/fpsyg.2022.1034872 36600710 PMC9807036

[9] Pino G, Amatulli C, Nataraajan R, De Angelis M, Peluso AM, Guido G. Product touch in the real and digital world: How do consumers react?. Journal of Business Research. 2020;112:492–501. doi: 10.1016/j.jbusres.2019.10.002

[10] Rathee R, Rajain P. Online shopping environments and consumer’s need for touch. J Adv Manag Res. 2019;16(5):814–26. doi: 10.1108/JAMR-12-2018-0116

[11] Shaban A, Saraeva A, Rose S, Clark M. The invisible hand of touch: Testing a tactile sensation‐choice satisfaction model in online shopping. J Sens Stud. 2024;39(1):e12897. doi: 10.1111/joss.12897

[12] Wang S, Ye Y, Ning B, Cheah J-H, Lim X-J. Why Do Some Consumers Still Prefer In-Store Shopping? An Exploration of Online Shopping Cart Abandonment Behavior. Front Psychol. 2022;12:829696. doi: 10.3389/fpsyg.2021.829696 35126270 PMC8811303

[13] Jiang K, Luo S, Zheng J. Seeing as Feeling? The Impact of Tactile Compensation Videos on Consumer Purchase Intention. Behav Sci (Basel). 2024;14(1):50. doi: 10.3390/bs14010050 38247702 PMC10813092

[14] Park J. On-line and off-line evaluation of roughness for cotton fabrics using quad analysis method. Seoul: Yonsei University. 2006.

[15] The Texture Institute. Textile terms and definitions. 6th ed. The Texture Institute. 1970.

[16] Haghzare L, Ping X, Arnison M, Monaghan D, Karlov D, Honson V, et al. Digital fabrics for online shopping and fashion design. Front Virtual Real. 2023;4. doi: 10.3389/frvir.2023.1236095

[17] Jeon D. Effects of haptic imagery on perceived quality and purchase intention by visual cues. Seoul: Seoul National University. 2022.

[18] Lee JM, Kim HY. Study on sensibility to visual tactility of modern fashion materials. J Basic Des Art. 2016;1017(5):447–60.

[19] Shaban A. The invisible hand of touch: Testing a tactile sensation-choice satisfaction model in online shopping. 2023. 10.1111/joss.12897

[20] Ezazshahabi N, Tehran MA, Latifi M. Predictive model for the frictional characteristics of woven fabrics optimized by the genetic algorithm. J Text Inst. 2018;109(8):1083–90. doi: 10.1080/00405000.2017.1400901

[21] Matusiak M, Kosiuk G. Quantitative Assessment of Woven Fabric Surface Changes During Martindale Abrasion Using Contactless Optical Profilometry. Materials (Basel). 2025;18(15):3636. doi: 10.3390/ma18153636 40805514 PMC12348827

[22] Lee H-T, Kim J-Y, Bak K, Kim K, Chun S, Hwang H-J. Predicting human tactile smoothness/roughness perception from multidimensional mechanical properties of synthetic fibers using machine learning. Sci Rep. 2025;15(1):42345. doi: 10.1038/s41598-025-26294-5 41309691 PMC12660992

[23] Begum MS, Milašius R. Factors of weave estimation and the effect of weave structure on fabric properties: a review. Fibers. 2022;10(9):74. doi: 10.3390/fib10090074

[24] Ezazshahabi N, Latifi M, Madanipour K. Modelling of surface roughness based on geometrical parameters of woven fabrics. Indian J Fibre Text Res. 2017;42(1):43–50.

[25] Beyene KA, Gebeyehu EK, Adamu BF. The effects of pretreatment on the surface roughness of plain-woven fabric by the Kawabata Evaluation System. Textile Research Journal. 2023;93(9–10):2149–57. doi: 10.1177/004051752211393

[26] Militký J, Mazal M. Image analysis method of surface roughness evaluation. Int J Cloth Sci Technol. 2007;19(3/4):186–93. doi: 10.1108/09556220710741650

[27] Park KH, Kwon YH, Oh KH, Kim EA. Measurement and analysis of surface roughness by a non-contact method for objective assessment of fabric handle. In: Proceedings of the Spring Conference of the Korean Society for Emotion and Sensibility, 2002. 357–60.

